# What elements of the work environment are most responsible for health worker dissatisfaction in rural primary care clinics in Tanzania?

**DOI:** 10.1186/1478-4491-12-38

**Published:** 2014-08-03

**Authors:** Godfrey M Mbaruku, Elysia Larson, Angela Kimweri, Margaret E Kruk

**Affiliations:** 1 Ifakara Health Institute, PO Box 78 373, Dar es Salaam, Tanzania; 2 Mailman School of Public Health, Columbia University, 722 West 168th Street, Room 603, 10032 New York, NY, USA

**Keywords:** Health systems, Job satisfaction, Rural healthcare, Tanzania

## Abstract

**Background:**

In countries with high maternal and newborn morbidity and mortality, reliable access to quality healthcare in rural areas is essential to save lives. Health workers who are satisfied with their jobs are more likely to remain in rural posts. Understanding what factors influence health workers’ satisfaction can help determine where resources should be focused. Although there is a growing body of research assessing health worker satisfaction in hospitals, less is known about health worker satisfaction in rural, primary health clinics. This study explores the workplace satisfaction of health workers in primary health clinics in rural Tanzania.

**Methods:**

Overall, 70 health workers in rural Tanzania participated in a self-administered job satisfaction survey. We calculated mean ratings for 17 aspects of the work environment. We used principal components analysis (PCA) to identify groupings of these variables. We then examined the bivariate associations between health workers demographics and clinic characteristics and each of the satisfaction scales.

**Results:**

Results showed that 73.9% of health workers strongly agreed that they were satisfied with their job; however, only 11.6% strongly agreed that they were satisfied with their level of pay and 2.9% with the availability of equipment and supplies. Two categories of factors emerged from the PCA: the tools and infrastructure to provide care, and supportive interpersonal environment. Nurses and medical attendants (compared to clinical officers) and older health workers had higher satisfaction scale ratings.

**Conclusions:**

Two dimensions of health workers’ work environment, namely infrastructure and supportive interpersonal work environment, explained much of the variation in satisfaction among rural Tanzanian health workers in primary health clinics. Health workers were generally more satisfied with supportive interpersonal relationships than with the infrastructure. Human resource policies should consider how to improve these two aspects of work as a means for improving health worker morale and potentially rural attrition.

**Trial registration:**

(ISRCTN 17107760)

## Background

Health workers in rural health facilities in low-income countries are crucial to addressing high maternal and newborn morbidity and mortality [1]. Their work is demanding and they have limited resources and support [2]. Tanzania, a low-income country in East Africa, has high maternal mortality and few health workers. The WHO estimated the maternal mortality ratio to be 410 maternal deaths for every 100,000 live births and the newborn mortality to be 41 per 1,000 live births [3,4]. In 2012, there were approximately 5 physicians and 50 nurses and midwives per 100,000 population, compared with the WHO minimum threshold of 228 health workers per 100,000 population [5-7]. Rural areas, where 70.4% of the population live, are particularly underserved [6,8].

This dearth of rural clinicians in the context of a resource-constrained health system can lead to varying degrees of dissatisfaction among health workers, manifesting as absenteeism, attrition, and labor unrest [9-11]. Thus, dissatisfaction exacerbates shortages and perpetuates the cycle of understaffing. Provider dissatisfaction may also be transmitted to patients through impatient, uncaring, or discourteous behavior. Previous research has found that women strongly value a positive health worker attitude when selecting their delivery facility [12].

In many countries in Africa and other low-income regions, health system policies have focused on expanding primary care in order to increase coverage of basic health services [13,14]. In Tanzania, the primary care system is extensive, with most people living within 10 km of a clinic [15]. This means that many of the primary care clinics are rural and difficult to access from the regional capital. They are staffed by lower cadres of health workers: clinical officers, nurses, and medical attendants, and are expected to provide basic preventative and curative services [14]. Clinical officers are non-physician clinicians who receive 3 years of clinical training following completion of 4 or 6 years of secondary school. Nurses have completed 4 years of secondary school and a subsequent 3 years of professional training. Medical attendants have the least training: they are generally primary school leavers (7 years of education) who receive 1 year of basic nursing training [16,17]. These primary care clinics (dispensaries) create the cornerstone of the healthcare system, however, they tend to be under-staffed and under-resourced [18].

Given the difficulties in recruiting and retaining rural health workers, and the link between health worker satisfaction and retention, it is essential to understand what influences health worker satisfaction and which elements may be amenable to policy change [5,19-21]. While there is a growing body of research on health worker satisfaction in rural health centers and hospitals, less work has been done examining health workers in the lowest level facility in sub-Saharan Africa [19,22,23]. Prior research has identified both intrinsic and extrinsic factors associated with health worker job satisfaction, including pay, management, availability of equipment, staffing and workload, and health worker age and cadre [22-24]. Recent literature has stressed the importance of the local context on the determinants of health worker job satisfaction [19,21]. Starting with previously identified drivers of satisfaction, this study seeks to assess the current level of satisfaction with aspects of the work environment among health workers in primary care clinics in rural Tanzania and identify areas where improvements may substantially affect general job satisfaction. Improving satisfaction may, in turn, lead to greater retention of rural providers.

## Methods

### Sample

**Figure 1:**
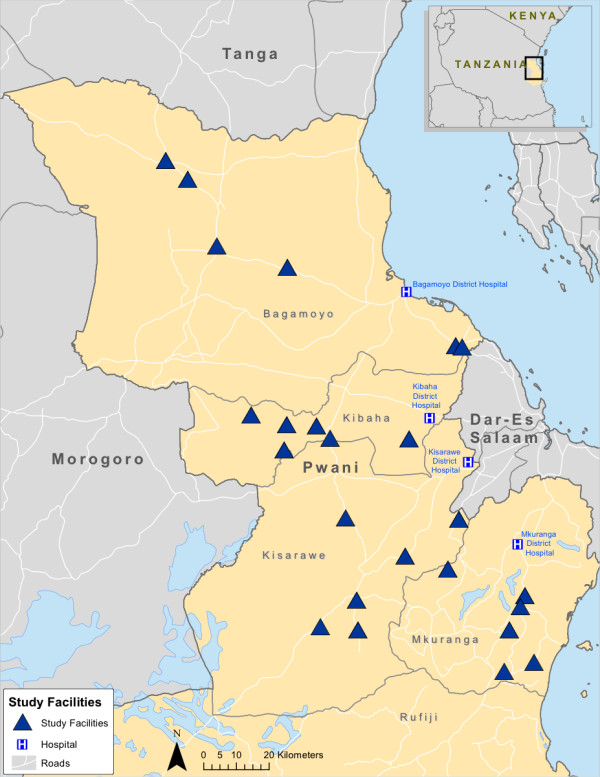
Map of study facilities, Pwani Region, Tanzania.

This analysis was conducted within a large cluster-randomized maternal and newborn health quality improvement study (ISRCTN 17107760). 24 government-managed primary healthcare facilities (dispensaries), in four districts of rural Pwani Region, Tanzania (Bagamoyo, Kibaha Rural, Kisarawe, and Mkuranga) were selected for inclusion in the study (Figure 1). To be included, facilities had to be government-managed, have at least one medically trained staff member (e.g., clinical officer or nurse), and be actively providing delivery services. From the 62 eligible facilities, the 6 primary care clinics from each district with the highest volumes of deliveries in the 6-month period from January to June 2011 were selected for inclusion.

All health workers working in the study facilities were invited to participate in the satisfaction survey. If health workers were not available on the day of data collection, the study team arranged for an additional visit to the facility in order to increase participation.

### Data collection

The self-administered survey was adapted from the Revised Nursing Work Index [25]. The survey includes a 17-question index addressing aspects of the work environment, asking health workers to state their agreement on a 4-point Likert scale. Additionally, we conducted an assessment of the 24 primary care clinics where the health workers were employed. This was done using a structured questionnaire adapted from the needs assessment created by the Averting Maternal Death and Disability Program and the UN system that has been previously used in more than 30 countries, including Tanzania [26]. The survey included questions regarding human resources, infrastructure, and services available as well as a record review of the volumes of services provided and was answered by the most senior provider available on the day of data collection.

Data were collected from December 5, 2011, to May 15, 2012, by three teams of three data collectors. Data collectors were trained for one week in ethical data collection and conducting interviews. The project was approved by ethical review committees at Columbia University (United States), Ifakara Health Institute (Tanzania), and National Institute for Medical Research (Tanzania). Written consent was obtained from each participant.

### Measures

The survey administered to health workers included 17 questions related to aspects of the work environment. An additional three questions related to overall job satisfaction were asked, namely, how satisfied they were with their own job, how satisfied other people are in similar jobs, and, finally, how much they would like to continue to work for this facility.

Based on the literature we identified a range of determinants of health worker satisfaction: health worker demographics, health worker length of employment, facility size, facility infrastructure, and services provided by the facility. We assessed health worker’s age, sex, cadre, and whether they were full or part-time. We categorized the time the health worker had worked in the facility at more than two years based on an inflection point seen in the data. To assess the workload of the facility we looked at the average monthly facility deliveries in 2011 and the average monthly outpatient visits for 2011. Where data were missing for a month in a facility we substituted the average of the contiguous months (data were missing for 2.3% of facility-months). If data were missing from two consecutive months (two instances), we created an average using the months where facility data were available. We also looked at the number of outreach visits the facility conducted in the 90 days before interview and the number of health workers at the facility. We assessed the facilities’ performance of basic emergency obstetric care (i.e., number of obstetric signal functions in the past three months, such as administering uterotonics or conducting newborn resuscitation), as a measure of the complexity of obstetric services provided in the facility. In order to assess the effect of the availability of equipment, supplies, and drugs on health worker satisfaction, we created an index. We utilized Tanzanian government guidelines, findings from the literature, and expert opinion to develop a list of essential obstetric equipment, supplies, and drugs [27,28]. We then calculated a summative score, where a single point was given for each item available and functioning on the day of assessment. We further assessed the infrastructure of the facility through the availability of electricity, clean water, and whether or not the facility had received an upgrade or renovation in the past year. We assessed the extent of supervision through the number of managerial meetings and supervisor visits in the past 90 days. Finally, because management of health facilities occurs through district-based teams, we assessed differences across the four districts in our study.

### Data analysis

Data were entered and variables were examined for missingness and outliers. Descriptive statistics were calculated for health worker and facility-level characteristics. We created a binary variable that grouped health workers into “strongly agree” versus all others, for health workers’ assessment of general job satisfaction.

We conducted a principal components analysis (PCA) of the 17 questions related to aspects of the work environment. PCA identifies underlying components that are described by the index through identifying questions that strongly correlate. Cattell’s Scree test graphically demonstrated that a majority of the variation in satisfaction was due to two components. We therefore extracted two components and used varimax rotation to simplify interpretation of the factors by allowing each variable to load strongly on only one factor. All 17 questions from the original index were maintained in the PCA. We created scores for each of the subscales using the sum of the subscale regression weights (factor loadings) multiplied by the healthcare worker’s response for each question [29]. We standardized the resulting subscales to aid in interpretation.

We calculated Cronbach’s α to assess internal consistency of the subscales. We then examined the association between the satisfaction subscale scores and each of the characteristics of the health workers and health facilities using bivariate linear regression analyses. We further explored correlation between dependent variables. Data analysis was conducted using Stata version 12.1 (StataCorp, 2012, College Station, TX, USA).

## Results

Of 100 eligible health workers, 70 participated in the survey (response rate = 70%). The 30 eligible health workers who did not participate were not in the facility on the day of interview. Of those who participated, 62 answered all 17 questions in the final satisfaction index. The health workers were primarily female (73%) and representative of the three cadres of workers generally seen in rural primary clinics in Tanzania: clinical officers, nurses, and medical attendants (Table 1). The average age of health workers was 40.8 years old. Nurses and medical attendants/maternal and child health aides (MCHA) were more likely than clinical officers to have worked in that facility for more than 2 years (88.9%, 63.0%, and 33.3%, respectively). Health workers in the study facilities averaged 0.53 in-service trainings per health worker in the first quarter of 2012, with 45.3% of health workers attending at least one training that quarter.

**Table 1:** Demographic characteristics of respondents (n = 70), Pwani Region, Tanzania

**Provider characteristics (n = 70)**	**% (n **^**ξ**^**)**	
Female	72.9 (51)	
Age (mean, SD)	40.8 (9.7)	
Cadre		
Clinical officer	30.0 (21)	
Nurse	25.7 (18)	
Medical attendant^Φ^	38.6 (27)	
Other	5.7 (4)	
Full time employment	94.3 (66)	
Worked in study facility for more than 2 years	60 (42)	
Education		
Diploma	30.4 (21)	
Certificate	60.9 (42)	
Other	8.7 (6)	
Received in-service training in past 90 days	45.3 (39)	
District of employment		
Bagamoyo	31.4 (22)	
Kibaha Rural	24.3 (17)	
Kisarawe	30.0 (21)	
Mkuranga	14.3 (10)	
**Facility characteristics (n = 24)**		
Workload		
Number of facility deliveries (mean, SD)	6.9 (4.9)	
Number of outpatient visits (mean, SD)	250.3 (117.7)	
Number of outreach visits in past 90 days (mean, SD)	3.3 (4.5)	
Number of healthcare workers at facility (mean, SD)*	4.2 (1.6)	
Renovation in the past year	25.0 (6)	
Equipment/supplies/medication index, max. 29 (mean, SD)	16.5 (3.6)	
Number of managerial meetings in past 90 days (mean, SD)	1.4 (1.4)	
Number of supervisor visits in past 90 days (mean, SD)	2.5 (1.8)	
Electricity	20.8 (5)	
Clean water	29.2 (7)	
BEmONC signal function index out of 7 (mean, SD)	2.1 (1.2)	

^ξ^Numbers may not add up to total as a result of missing values.

ΦIncludes medical attendants and maternal and child health aides.

*Data are for the 90 day period before the May 2012 interview. The health workers represented here are a proportion of all health workers in the facility, not limited to those who completed the satisfaction questionnaire.

The average number of deliveries conducted by the facility per month was 6.9, ranging from 0.9 to 22.8. Less than one third of facilities had electricity and clean water (20.8% and 29.2%, respectively). On average, the facilities had performed 2.1 out of 7 of the basic emergency obstetric signal functions in the prior 3 months (Table 1).

Most respondents strongly agreed that, in general, they are satisfied with their job (51 respondents, 73.9%). Fewer respondents (37 respondents, 54.4%) strongly agreed that most people in their job are very satisfied with it. Overall, 69.1% of respondents strongly agreed that they would like to continue working for their current health facility for quite some time.

Health workers tended to be more satisfied with the environment in which they worked than the tools of their job: 73.9% of health workers strongly agreed that they had the freedom to make important decisions and 67.6% felt that their opinions were respected, but only 15.9% strongly agreed that there were enough staff, only 2.9% strongly agreed that there was enough functioning equipment and infrastructure, and only 11.6% strongly agreed that they were satisfied with their pay compared to similar jobs in other organizations (Table 2).

**Table 2:** Mean satisfaction with 17 items of work environment

	**Providers who strongly agree, % (n **^**ξ**^**)**	**Providers who agree, % (n **^**ξ**^**)**
In general, I am satisfied with this job*	73.9 (51)	21.7 (15)
Freedom to make important patient care and work decisions	73.9 (51)	24.6 (17)
Adequate pre-service education for my current position	70.6 (48)	26.5 (18)
I find that my opinions are respected at work	67.6 (46)	26.5 (18)
Clinical officers, nurses and other health workers have good working relationships	66.7 (46)	29.0 (20)
Adequate in-service (continuing) education to improve my skills	66.7 (46)	26.1 (18)
Adequate access to referral to a higher-level facility for sick patients	59.4 (41)	29.0 (20)
I am satisfied with the recognition I get for the work that I do	55.9 (38)	38.2 (26)
District health managers support and value health workers	48.6 (34)	44.3 (31)
Adequate clinical supervision in this position	44.3 (31)	47.1 (33)
Adequate mentoring and support to assist me in this position	30.4 (21)	49.3 (34)
I feel that my workload is manageable (not too heavy)	27.5 (19)	44.9 (31)
Enough time and opportunity to discuss patient care problems with other staff	21.7 (15)	55.1 (38)
Enough staff to provide quality patient care	15.9 (11)	44.9 (31)
I am satisfied with my pay compared to similar jobs in other organizations	11.6 (8)	42.0 (29)
Consistent availability of supplies and medications to perform my duties	10.0 (7)	58.6 (41)
Enough staff to get the work done	7.1 (5)	50.0 (35)
Functioning equipment and infrastructure to perform my duties	2.9 (2)	62.9 (44)

^ξ^Numbers may not add up to total as a result of missing values.

*Measure of overall satisfaction, not included in the PCA analysis.

The PCA identified two dimensions of satisfaction within the 17-item satisfaction questionnaire. These subscales explain 45.4% of the variance in those 17 items (Table 3). Cronbach’s α was 0.8373 for the first subscale and 0.8087 for the second. The first subscale is related to the availability of adequate infrastructure and resources, or “tools to get the job done” and includes the providers’ satisfaction with factors such as availability of providers, equipment, drugs, and infrastructure. The second subscale is related to supportive interpersonal and mentoring environments and includes the providers’ opinions on adequate supervision, mentoring, and relationships between staff.

**Table 3:** Factor loadings for the two main subscales identified through principal component analysis of the 17-question health worker satisfaction survey (n = 62)

	**Factor loading**	
**Subscale 1: Tools to get the job done (explains 25.4% of overall variance)**		
Enough staff to provide quality patient care	0.4079	
Consistent availability of supplies and medications to perform my duties	0.3861	
Enough staff to get the work done	0.3835	
I am satisfied with my pay compared to similar jobs in other organizations	0.3829	
I feel that my workload is manageable (not too heavy)	0.3413	
Functioning equipment and infrastructure to perform my duties	0.2896	
Adequate pre-service (continuing) education to improve my skills	0.2209	
Enough time and opportunity to discuss patient care problems with other staff	0.1883	
**Subscale 2: Supportive interpersonal environment (explains 20.0% of overall variance)**		
Clinical officers, nurses and other health workers have good working relationships	0.4023	
I find that my opinions are respected at work	0.3895	
Freedom to make important patient care and work decisions	0.3873	
Adequate clinical supervision in this position	0.3328	
Adequate access to referral to a higher-level facility for sick patients	0.3289	
District health managers support and value health workers	0.3284	
Adequate mentoring and support to assist me in this position	0.2726	
I am satisfied with the recognition I get for the work that I do	0.2534	
Adequate in-service (continuing) education to improve my skills	0.2023	

Women scored higher than men, and nurses and medical attendants/MCHA scored higher than clinical officers on the supportive interpersonal subscale. More facility deliveries and a higher score on the basic emergency obstetric and newborn care (BEmONC) signal function index were associated with reduced supportive interpersonal satisfaction subscale scores. Increased age and being a nurse were both associated with increased satisfaction on the infrastructure subscale (Table 4). Age and the time the health worker had worked in their current facility had a correlation of 0.24.

**Table 4:** Bivariate associations between each of the satisfaction indices and provider and facility characteristics

**Provider characteristics**	**Infrastructure**	***P *** **value**	**Interpersonal**	***P *** **value**
	**Coefficient**		**Coefficient**	
Sex (female)	-0.06	0.839	0.60^*^	0.043
Age (continuous)	0.03^*^	0.035	0.02	0.084
Cadre (reference: clinical officer)				
Nurse	0.69^*^	0.040	0.73^*^	0.024
Medical attendant/MCHA	0.48	0.125	0.75^*^	0.015
Other	0.52	0.400	-0.34	0.571
Full time	-0.22	0.675	0.04	0.935
Worked in this facility for more than 2 years	0.55^*^	0.035	0.46	0.079
**Facility characteristics**				
Workload				
Average facility deliveries per month in 2011	-0.04	0.080	-0.05^*^	0.049
Average outpatient visits per month in 2011	0.002	0.132	0.001	0.228
Number of outreach visits in past 90 days^ξ^	0.02	0.525	-0.03	0.286
Number of healthcare workers at facility	0.13	0.072	0.03	0.733
Upgrade or renovation in past year	-0.004	0.989	0.08	0.801
Equipment/Supplies/Medication index	0.05	0.205	-0.04	0.307
Number of managerial meetings in past 90 days	0.07	0.349	0.03	0.690
Number of supervisor visits in past 90 days	0.02	0.793	0.06	0.382
Electricity available	-0.13	0.664	0.36	0.236
Clean water available	-0.23	0.416	-0.002	0.995
BEmONC signal function index out of 7	0.02	0.843	-0.33^*^	0.004
District (comparison is Bagamoyo)				
Kibaha rural	0.50	0.142	0.47	0.167
Kisarawe	0.61	0.062	0.05	0.871
Mkuranga	0.36	0.366	-0.56	0.154

^ξ^Data are for the 90 day period before the facility interview.

^*^P value <0.05.

## Discussion

In this study, we found that although health workers were satisfied with their jobs in general, they were much less satisfied with different elements of their work environment. Health workers were substantially more satisfied with the current status of their supportive interpersonal environment than they were with their pay or aspects relating to the infrastructure needed for good technical quality of care (e.g., drugs and equipment). The finding that substantial proportions of health workers report high satisfaction even in adverse environments has been reported in the literature [19,21]. The likely reasons for the high stated satisfaction include community appreciation, health worker expectations, and organizational structure [21,30,31].

Most health workers were satisfied with the level of training they received. In-service trainings were commonly attended by health workers in our study facilities and, in the Pwani Region, as in other areas, in-service training often provides benefits in addition to the skills being taught: health workers stationed in rural villages typically come to town for these trainings and receive a per diem payment or top-up pay covering their expenses. This, in addition to the skills enhancement offered by in-service training, may contribute to the popularity of in-service training among health workers [31,32].

In this study almost half of health workers were dissatisfied with their level of pay. Low salary levels have been previously reported to negatively influence job satisfaction and motivation [24,33,34]. There is currently a pay for performance scheme underway in the region under study and initial evidence suggests that the scheme may help motivate staff to reach targets [35]. However, systemic constraints were perceived as impediments to health workers and facilities reaching their targets [35]. Although health workers are dissatisfied with their pay, and the pay for performance scheme is a potential opportunity to increase their pay and to motivate them to meet specific targets, this will not work if improvements to infrastructure (also associated with health worker satisfaction) are not first improved.

Two main dimensions of satisfaction with work environment emerged through the PCA: adequate infrastructure and supportive interpersonal relationships. Previous research in other health care settings has found similar drivers of staff motivation [10,12,23,24,36]. Because health workers were generally dissatisfied with the level of resource availability and staffing numbers, there may be more opportunity for improvement in these areas. Health worker job satisfaction in performing specific tasks, such as providing care for malaria, has been shown to be related to in-service training, financial compensation, adequate resources, working relationships, and management support [11,24,31,33,34,36,37].

The bivariate analysis identified provider-level characteristics that were associated with satisfaction. As providers get older they become inured to working in low-resource environments, which may explain their increased satisfaction with infrastructure compared to their younger colleagues. We found health workers who have worked in the facility for longer than 2 years were more satisfied with the infrastructure than more recent arrivals. This may in part be because satisfied health workers stay longer, or health workers who have stayed longer are more inured to the actual working conditions in rural health facilities. It may also be because moving from one facility to another is disruptive and leads to reduced job satisfaction. Although other researchers have found a similar positive association between health worker age and job satisfaction [22], the cross-sectional design of this study does not allow us to determine the direction of causality for the positive correlation between age and time worked in the current facility that we found. Future research should explore this relationship further.

Clinical officers were less satisfied with infrastructure and supportive interpersonal environment than nurses or health aides. This may be because they are trained to provide a broader range of services than nurses and medical attendants, including diagnostic and treatment services [16]. Clinical officers’ reduced satisfaction may thus be the result of higher expectations for their care environment and their resulting frustration with the lack of infrastructure in the primary care clinics. These findings differ from those of McAuliffe et al. [23], whose survey of health workers in health centers and hospitals found that medical doctors were more satisfied with working relationships than their nursing counterparts. Their survey included slightly different questions in the index, but, more importantly, involved health workers in higher-level facilities. Viewing the two studies together elucidates the important role that the health facility environment may play in health worker satisfaction.

Health workers in facilities performing more deliveries were less satisfied with supportive interpersonal relationships, as were health workers in facilities performing more BEmONC signal functions. BEmONC signal functions are an indicator of the scope and complexity of delivery services a facility provides [26]. As these primary-care facilities get busier and provide more complex services, health workers may become more stressed and less happy with their team. Interestingly, no other facility-level factors were associated with satisfaction. This is probably due to a lack of heterogeneity in the facilities included in this study; they are all under-staffed and under-resourced.

This study has several limitations. The sample was small and not representative of the entire region. The study took place among health care workers in the six eligible dispensaries per district that had the most deliveries; health workers in dispensaries with even fewer deliveries per month may have different determinants of workplace satisfaction. Data on the eligible providers who were not present for interview are not available and these providers may differ from those interviewed. Two of the variables measuring provider workload, number of facility deliveries and number of outpatient visits, were routine facility service statistics which are often subject to higher missingness and inaccuracy than data collected directly by researchers. However, these variables are yearly averages and are probably not subject to enough error to significantly impact the results. Social desirability may have biased health workers’ responses, as suggested by the more positive responses given to the question asking if they themselves are satisfied with their job, as compared to if they think most people in the same position are satisfied. Finally, this study took place among rural health workers, whose experiences differ substantially from those in more urban areas.

## Conclusions

Health system improvements should target areas that will most improve health worker’s general job satisfaction. Job satisfaction is associated with intent to stay, and health worker retention is necessary for the provision of high quality healthcare [19]. The results from this study suggest that effort should focus on providing healthcare workers with the infrastructure and tools they need to do their jobs. This includes sufficient staff, equipment, supplies, medicines, and general infrastructure. In addition to being necessary for health workers to deliver high quality care, this could foster health worker satisfaction, increasing the benefits of the investment. This is important to consider in the context of the many ongoing experiments with pay for performance, which are unlikely to boost satisfaction if weak infrastructure prevents health workers from successfully fulfilling their tasks.

## Abbreviations

BEmONC: Basic emergency obstetric and newborn care; MCHA: Maternal and child health aides; PCA: Principal components analysis.

## Competing interests

The authors declare that they have no competing interests.

## Authors’ contributions

GMM contributed to the study design and protocol development, oversaw interpretation, and edited the manuscript. EL oversaw data collection, conducted data analysis, and drafted the manuscript. AK oversaw data collection and edited the manuscript. MEK contributed to the study design and protocol development, oversaw data analysis and interpretation, and edited the manuscript. All authors read and approved the final manuscript.
